# Role of Child Welfare in Detection and Treatment of Early Childhood Developmental Concerns

**DOI:** 10.1001/jamahealthforum.2025.4554

**Published:** 2025-10-24

**Authors:** Christian M. Connell, Ezra G. Goldstein, Ziyu Huang, Sarah A. Font

**Affiliations:** 1 Department of Human Development and Family Studies, The Pennsylvania State University, University Park; 2 Child Maltreatment Solutions Network, Social Science Research Institute, The Pennsylvania State University, University Park; 3 Jimmy and Rosalynn Carter School of Public Policy, Georgia Institute of Technology, Atlanta; 4 Brown School, Washington University in St Louis, St Louis, Missouri

## Abstract

This cohort study assesses the associations of child welfare system responses with developmental disorders and early intervention services.

## Introduction

Children benefit from early identification and treatment of developmental disorders (DDs) or missed milestones.^1^ Young children involved with the child welfare system (CWS) face increased risk of DDs due to biological risk factors and maltreatment^2^; thus, DD rates are elevated in this population.^3^ Higher diagnosis rates may also reflect increased clinical scrutiny or diagnostic bias after CWS involvement—particularly in foster children. The Child Abuse Prevention and Treatment Act requires states to refer young children with substantiated maltreatment to early intervention (EI) services. Although EI services may improve developmental outcomes,^4^ many eligible children are not referred.^5^ Our aim was to evaluate EI service use associated with 3 CWS responses.

## Methods

The Pennsylvania State University Institutional Review Board approved this cohort study and waived informed consent because the study used existing records, did not involve human participants, and posed only minimal risk. We followed the STROBE reporting guideline.

We used linked data from the Pennsylvania Child Welfare Information System and Medicaid for children born between 2015 and 2017 with Medicaid claims between 2015 and 2019. Independent variables were CWS response type: no services (substantiated), in-home services, or foster care. Dependent variables included DD diagnosis, well-child visits, and EI service use. Covariates included child demographics, CWS allegations, and maternal history of substance use disorder and serious mental illness (eMethods in Supplement 1).

Stacked difference-in-differences (SDID) models^6^ compared outcomes among children aged 3 years or younger in the 3 CWS response groups and a reference group of children not yet involved with CWS who had a subsequent referral. We estimated temporal differences among groups, assuming outcomes would follow trends similar to those without CWS contact. Additional analyses examined potential mechanisms of the main findings. Data were analyzed from March 2024 to December 2024 using Stata 18 (Stata Corp).

## Results

The sample included 10 368 referrals (22 549 children; mean [SD] age, 1.5 [0.7] years; 4954 females [47.8%] and 5414 males [52.2%]): 5940 for no services, 3512 for in-home services, and 916 for foster care. Among 18 235 children in the control group, 32% had a DD diagnosis during the study period. DD rates within 12 months following CWS contact were higher for children with confirmed CWS referrals: 36% for no service, 45% for in-home services, and 63% for foster care, largely due to missed milestones and unspecified delays.

**Figure 1.**
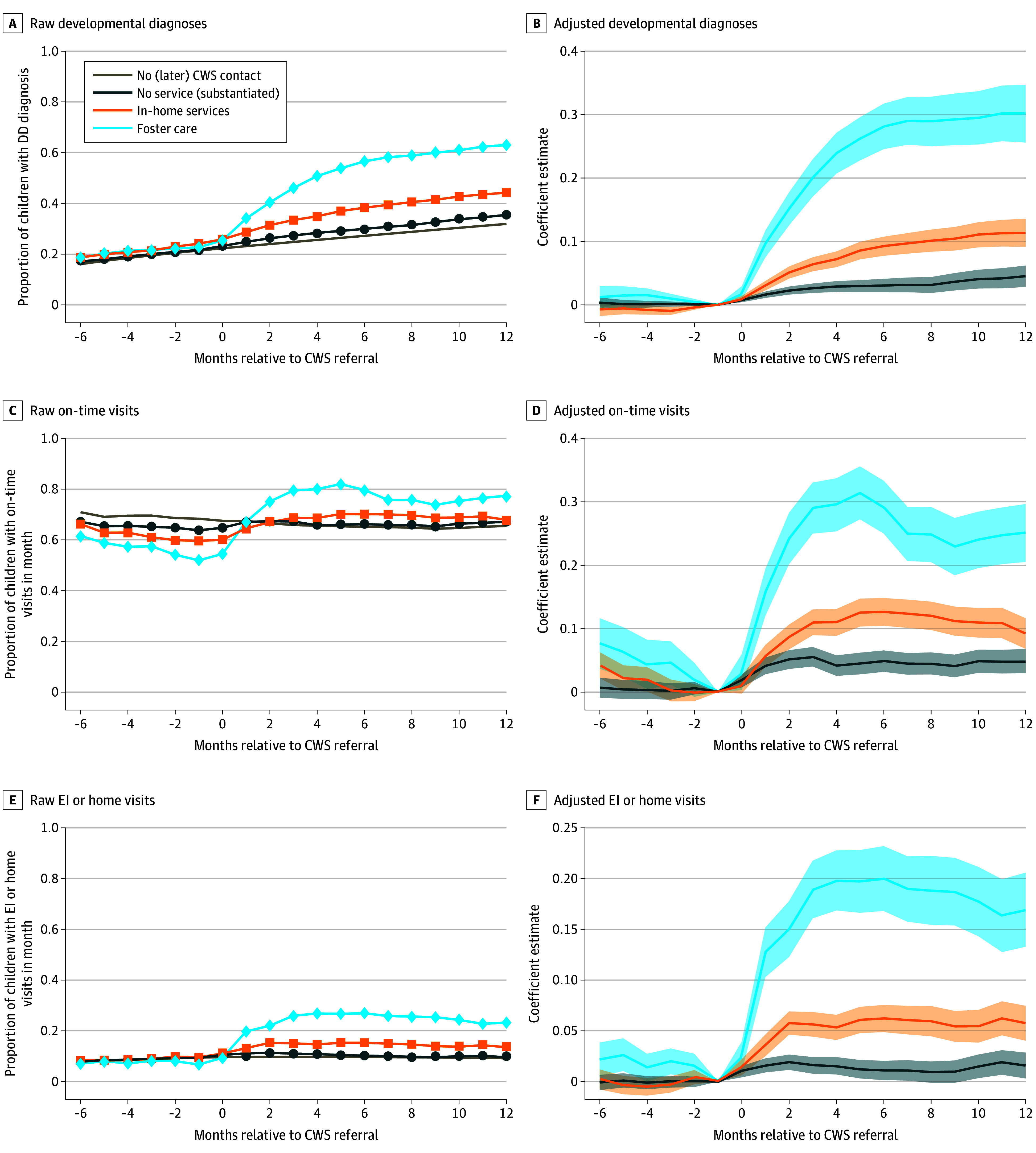
Associations of Child Welfare System (CWS) Response With Developmental Disorder (DD) Diagnosis, On-Time Well-Child Visits, and Early Intervention (EI) Service Use Regression-adjusted estimates omit the month prior to referral, and scaled to the mean of the no (later) CWS contact group in that month, with the y-axis showing percentage-point differences relative to that group. Shaded areas represent the 95% CIs.

Regression-adjusted SDID estimates show a 30–percentage point (pp) increase in DD diagnosis after referral the foster care, with 10-pp and 5-pp increases for in-home services and no-service, respectively (Figure 1). On-time well-child visits and EI service use had the largest increases among the foster care group (30 pp and 20 pp, respectively), with smaller increases in the other 2 groups.

**Figure 2.**
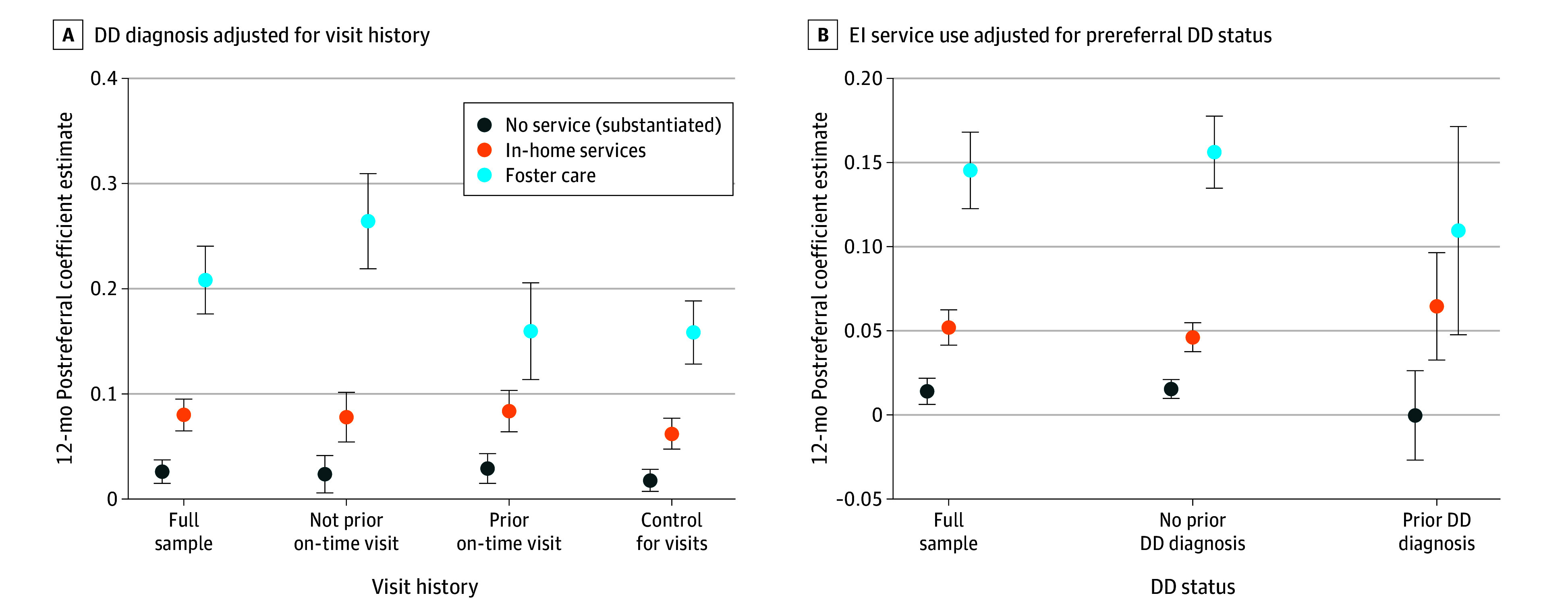
Adjusted Stacked Difference in Differences (SDID) Estimates of the Associations of On-Time Well-Child Visits With Developmental Disorder (DD) Diagnosis and DD Status With Early Intervention (EI) Service Use The plots show the postreferral mean SDID estimates over 12 months. Error bars represent the 95% CIs.

Associations between increased DD diagnosis and improved access were attenuated (12 month postreferral average: 16 pp vs 21 pp) (Figure 2). Results also showed elevated but attenuated EI use for foster care (11 pp vs 14 pp) among youth with prior DD diagnosis, suggesting active CWS involvement may facilitate access beyond DD diagnosis.

## Discussion

All 3 CWS response types were associated with increased DD diagnosis and EI service use in children aged 3 years or younger, with the largest increase for foster care placement. The immediacy of increases in diagnosis, coupled with follow-up analysis of other preventive care services, suggests that foster care is not increasing DDs but rather diagnosis and treatment. Foster care increases compliance with timely well-child visits, where detection of preexisting DDs may occur. Additionally, DD diagnosis increased immediately following foster care entry even for children with a well-child visit shortly before entry, suggesting that clinicians may differentially diagnose DDs in this population. Finally, increases in EI service use were larger for children entering foster care regardless of prior diagnostic status, which may reflect heightened CWS efforts to promote, mandate, or facilitate service for children in their custody.

Study limitations include the focus on a single state, limiting generalizability, and inability to assess referrals not resulting in service uptake. Thus, selective uptake by caregivers vs differential efforts by agencies or clinicians to connect families to services could not be assessed.

## Notes

Supplement 1.
eMethods

eReferences

Supplement 2.
Data Sharing Statement
