# Supplementary material for: Role of Child Welfare in Detection and Treatment of Early Childhood Developmental Concerns
Source: JAMA Health Forum. 2025 Oct 24;6(10):e254554. doi: 10.1001/jamahealthforum.2025.4554 (PMC12552921; doi:10.1001/jamahealthforum.2025.4554)
Supplement: Supplement 1. — eMethods eReferences [file jamahealthforum-e254554-s001.pdf]

# Supplemental Online Content

Connell CM, Goldstein EG, Huang Z, Font SA. Role of child welfare in detection and treatment of early childhood developmental concerns. *JAMA Health Forum*. Published online October 24, 2025. doi:10.1001/jamahealthforum.2025.4554

**eMethods**

**eReferences**

This supplemental material has been provided by the authors to give readers additional information about their work.

© 2025 Connell CM et al. *JAMA Health Forum*.

**eMethods**

## Study Approval and Data Security

This study was reviewed by the Pennsylvania State University IRB. Data were obtained through a Business Associate Agreement between the university and the state's Child Welfare and Medical Assistance agencies and were maintained in a secure computing environment with ongoing monitoring to ensure compliance with an approved data security plan.

## Study Population and Participants

The sample included probabilistically linked administrative data from Pennsylvania's Child Welfare Information System (CWIS) and the Office of Medical Assistance Programs (Medicaid) for children with a history of confirmed CWS involvement at some point. The sample was restricted to children born between 2015 and 2017 who had Medicaid claims between the ages 0 to 3. CWIS data was used to identify all confirmed CWS cases for children before age 3 occurring between 2015 and 2019 (N=25,329 referrals involving 26,475 children), and data were linked with statewide Adoption and Foster Care Reporting and Analysis System records to identify CWS cases resulting in foster care placement within 90 days of referral. We excluded cases involving children with no evidence of Medicaid claims in the 6 months prior to and 12 months following a CWS referral (6528 referrals).

The final analytic sample consisted of 23,671 CWS child-referrals (n=22,549 children). The exposure group included 10,368 CWS child-referrals (n=10,066 children). Of child-referrals, 5940 received no formal CWS service response, 3512 were accepted for in-home services, and 916 were placed in foster care within 90 days of the referral. Most children experienced one CWS case referral before age 3 (97.1%, n=10,066); 2.9% (n=302) had two CWS referrals eligible for study inclusion. A total of 18,235 children were included in the comparison group.

© 2025 Connell CM et al. *JAMA Health Forum*.

This group included children who subsequently entered the exposure group prior to age 3 but were enrolled in Medicaid and supplied data on healthcare outcomes prior to CWS contact (n=5752), as well as children with Medicaid claims who did not experience a confirmed case prior to age 3—but did experience a confirmed CWS case after age 3 (n=12,483). Sample characteristics for the CWS exposure groups are summarized in eTable 1.

**eTable 1. Sample Characteristics at child-referral level (%).**

| | Substantiated Only | In-Home Services | Foster Care Placement |
|---|---|---|---|
| Number of child-referrals | 5,940 (57.3%) | 3,512 (33.9%) | 916 (8.8%) |
| **Child Age at CPS referral** | 1.56 | 1.48 | 1.41 |
| **Child Race/Ethnicity** | | | |
| White | 0.68 | 0.62 | 0.58 |
| Black | 0.18 | 0.22 | 0.30 |
| Hispanic | 0.10 | 0.12 | 0.10 |
| Other race/ethnicity | 0.04 | 0.03 | 0.02 |
| **Child Sex (Male)** | 0.52 | 0.52 | 0.54 |
| **Developmental Diagnoses (Prior to CWS Referral)** | | | |
| Any Developmental Diagnosis | 0.22 | 0.24 | 0.23 |
| Autism or PDD | 0.00 | 0.00 | 0.00 |
| Speech/Language Delay | 0.01 | 0.01 | 0.01 |
| Cognitive Delay | 0.00 | 0.00 | 0.00 |
| Motor Delay | 0.01 | 0.00 | 0.01 |
| Growth Delay | 0.05 | 0.07 | 0.06 |
| Milestone or Other Developmental Delay | 0.18 | 0.20 | 0.19 |
| **CPS Referral** | 0.04 | 0.04 | 0.13 |
| **CPS Allegations** | | | |
| Parent substance use/prenatal exposure | 0.45 | 0.44 | 0.46 |
| Unmet material needs | 0.29 | 0.37 | 0.32 |
| Failure to protect | 0.24 | 0.23 | 0.25 |
| Parent mental illness or disability | 0.14 | 0.17 | 0.15 |
| Inadequate supervision | 0.12 | 0.11 | 0.14 |
| Inappropriate caregivers | 0.09 | 0.10 | 0.10 |
| Inappropriate discipline/corporal punishment | 0.05 | 0.04 | 0.02 |
| Any physical abuse | 0.03 | 0.03 | 0.08 |
| Abandonment or lack of caregiver | 0.02 | 0.02 | 0.07 |
| Emotional maltreatment | 0.02 | 0.04 | 0.03 |
| Child health or conflict with parent | 0.01 | 0.01 | 0.00 |

© 2025 Connell CM et al. *JAMA Health Forum*.

| | | | |
|---|---|---|---|
| Any sex abuse | 0.00 | 0.00 | 0.00 |
| Moral neglect | 0.00 | 0.00 | 0.00 |
| **Maternal Primary Substance Use Diagnosis (Prior to CWS Referral)** | 0.40 | 0.52 | 0.67 |

**Measures**

Independent variables. The CWS response to individual child-case referrals were categorized as: (1) confirmed CWS with no service response [substantiated only], (2) confirmed CWS with in-home services [in-home services], or (3) confirmed CWS with foster care placement within 90 days [foster care placement]. We identified the first two groups based on case disposition in CWIS records, and the third group was based on foster care placement within 90 days using linked foster care records. The fourth group—later CWS comparison cases—comprises children who met age-based criteria and Medicaid record inclusion criteria (i.e., annual claims in birth-to-three period) but had not yet experienced a confirmed CWS referral during the birth-to-three observation window, though all did have a subsequent confirmed referral to CWS.

Dependent variables. Medicaid claims from 2015-2020 were used to create indicator variables identifying person-months for which a child-referral was involved in at least one claim involving an outcome of interest. We identified timing of inpatient, outpatient, or professional claims involving a primary or secondary DD diagnosis based on established ICD-9 or ICD-10 criteria for presence of a pervasive developmental disorder or delay in speech/language, cognitive, motor, growth, or milestone and other developmental delays (e.g., delayed milestones, other or unspecified delays)[1]. Medicaid Current Procedural Terminology (CPT) codes were used to identify receipt of on-time well-visit claims based on timing relative to child's age and recommended frequency of preventive medical care services (see eTable 2). Finally, early-intervention or home visiting (EI/HV) claims were based on provider codes included in claims

© 2025 Connell CM et al. *JAMA Health Forum*.

data (see eTable 3).

**Supplement Table 2. Codes for On-time Well-Visits**

| Type of Services | CPT Codes | ICD-10 Codes | ICD-9 Codes |
|---|---|---|---|
| On-time Well-visits | 99460, 99463, 99381, 99382, 99391, 99392, 96110, 99349, 99341, 99600, 99501, 99348, 99502, 99350, 99349, 99341, 99600, 99501, 99348, 99502, 99350 | Z00.110, Z00.8, Z00.111, Z00.121, Z00.129, Z02.89 | V20.2, V20.3, V70.0, V70.3, V70.5, V70.6, V70.8, V70.9 |

**Supplement Table 3: Provider Codes for EI/HV Services**

| Type of Services | Provider Codes |
|---|---|
| EI/HV Services | Early Intervention Case Management, Early Intervention Services, EI Preschool Provider, Licensed Social Worker/EI, Nurse Family Partnership, Nutritional Support/ Early Intervention, Occupational Therapy/ Early Intervention, Physical Therapy/ Early Intervention, Preschool Early Intervention Occupational Therapy, Preschool Early Intervention Physical Therapy, Preschool Early Intervention Service, Special Instruct-Behavior/Early Intervention, Speech/Hearing Therapy/ Early Intervention |

Our primary outcomes focus on early childhood health care utilization—including adherence to the recommended well-child visit schedule during infancy and early childhood, as well as use of EI/HV services. Well-visit scheduling guidelines, as outlined by the American Academy of Pediatrics, specify a series of closely spaced well-child visits in the first years of life (at 2, 4, 6, 9, and 12 months, for example) with the intent of monitoring growth, administering vaccines, and identifying developmental concerns early.

To align with these standards, our measure of timely visitation assesses whether children received subsequent well-visits within appropriate time windows. For instance, among children who had a 2-month visit, we examine whether they received another well visit within the next two months, consistent with the expected schedule. At most, children who had a visit by age 2

© 2025 Connell CM et al. *JAMA Health Forum*.

years, should have a subsequent well-child visit by age 3 years.

Covariates. Child race/ethnicity, sex, and CPS allegation were derived from CWIS data. Maternal history of substance abuse diagnosis and serious mental illness were derived from Medicaid claims data. We also included fixed effects to account for the year and referral county.

**Statistical Analysis**

To examine how CWS contact affects the identification of DD and the provision of EI services, we implemented stacked differences-in-differences (SDiD) models[2,3]. These models compared changes in DD diagnoses or service outcomes before and after a focal CWS referral and between several types of formal CWS contact responses (three exposure groups) and those who had not yet encountered CWS (unexposed group), while accounting for a rich set of covariates. All analyses were conducted using Stata 18.5[4].

We view the use of a “not-yet-exposed” comparison group as a strength rather than a limitation. Children who eventually experience CWS involvement are more comparable in underlying risk to those with early referrals than children never referred, who may differ systematically in unobserved ways. This approach improves internal validity by providing comparisons among similarly at-risk populations, yielding more credible estimates of how CWS response timing influences identification of developmental diagnoses during early childhood and service access. Of course, this results in a trade-off of external validity to other children (e.g., those who never encounter CWS), which we discuss as a general limitation.

Key variables of interest included interaction terms between indicators for the month relative to a CWS referral (6 months prior to 12 months post) and indicators for CWS responses (substantiation only, in-home services, and foster care placement). Standard errors were clustered by child, and $p \leq 0.05$ was considered as statistically significant. For ease of interpretation, we

© 2025 Connell CM et al. *JAMA Health Forum*.

estimated our model via multivariable linear regression so that estimates could be interpreted as percentage point changes relative to the comparison condition.

A known challenge with the standard DiD design is the potential for biased estimates when comparing units receiving treatment at different time periods[5]. Specifically, the bias arises from comparing treated units at different stages of treatment, leading to problematic comparisons if the effects of the treatment are not constant. To address this, we implemented an SDiD design, which mitigates this concern[2,3]. The SDiD approach ensures comparisons between treatment units and "clean control" units, i.e., those who have not yet been treated. This is done by creating a series of treatment-event-specific datasets.

In our context, a cohort is formed that includes pre- and post-treatment observations for children who come into contact with CWS in a given month (*t*), as well as children who have not yet come into contact with CWS by that time. Crucially, children who have already been treated by month (*t*) (i.e., already treated units) are excluded. This leaves a dataset composed only of children who were either newly treated in that month or were still untreated. For each month in which a CWS referral occurs, we separately construct an analogous dataset.

We then "stack" these focal referral month datasets in relative time, effectively mimicking a scenario where all CWS contact events occur contemporaneously. This ensures that comparisons are made between treated units and valid control units, avoiding bias from staggered treatment timing. Using the “stack” as our analysis sample, we then estimate the following event-study specification:

$$ChildOutcome_{it} = \sum_{j \neq -1} FosterCare_i \gamma_j + \sum_{j \neq -1} \text{Services}_i \delta_j + \sum_{j \neq -1} Substantiated_i \sigma_j + \mathbf{X}'_{it} \beta + \varepsilon_{it} \quad (1)$$

For each child outcome, our unit of interest is an individual child (*i*) involved in a CWS referral observed in a given month (*t*) relative to the beginning of a referral (ranging from 6-

© 2025 Connell CM et al. *JAMA Health Forum*.

months prior to the referral and 12 months post referral). Child outcomes are indicator variables coded to be equal to one if the individual child experienced the event (e.g., diagnosis, well-visit, EI service, etc.) in the given month. The independent variables *FosterCare, Services, and Substantiation* identify children on CWS referral with a specific type of CWS contact, either foster care placement, in-home services, or substantiation only, respectively. The specification includes separate indicators for 6 lags and 12 leads relative to the beginning of a CWS referral and for the type of formal CWS response. This allows the model to flexibly estimate changes in DD diagnoses or EI services relative to the month prior to the referral and relative to children without CWS contact in the same month. Finally, $X_{it}$ is a vector of controls including child race/ethnicity, sex, CPS allegation, maternal history of substance abuse diagnosis and serious mental illness, as well as referral year and county. Standard errors are clustered at the child-level to account for arbitrary correlation across referrals that involve the same child. Following Wing et al, we additionally apply corrective sample weights corresponding to the size of each "stack."[1]

A fundamental assumption of our approach is that the identification of DD or provision of EI services among children who had contact with CWS would have evolved similar to those without contact had a referral not occurred[2,3,5,6]. This assumption implies that the gamma, delta, and omega *js* should be equal to zero for all $j \leq -1$. Model results show small, though statistically significant, deviations from zero among certain CWS groups in the pre-referral period. Two sets

[1] Wing et al show that a basic stacked estimator does not identify the target aggregate causal effect due to implicit weights applied to treatment and control trends. Instead, they suggest a corrective weighting procedure that eliminates the implicit weighting bias and produces an unbiased estimate of the target parameters.
In some models, the outcome of interest is the cumulative incidence of DD diagnosis or EI services. In these models, we employ the above specification with two modifications. First, the outcome is instead the ever-to-date prevalence of the outcome of interest by month *t*. Second, cumulative models account for linear group-specific trends, represented by interactions between the CWS response type indicators and the calendar year-month combination of the observation.

© 2025 Connell CM et al. *JAMA Health Forum*.

of additional analyses were conducted to probe for potential mechanisms underlying the main findings. First, to assess whether increased DD diagnoses reflected improved access to health care, the SDiD DD identification model was re-run, stratifying the sample by pre-referral well-visit status (i.e., for groups with or without prior on-time well-visits), as well as a second model that adjusted for the post-referral medical visit occurrence. Second, to assess whether elevated EI service use was associated primarily with the occurrence of newly identified versus pre-existing DD concerns, the SDiD EI model was re-run, stratifying the sample by pre-referral presence of a prior DD diagnosis (i.e., for groups with or without a prior DD diagnosis). Figure 2 plots in the primary article depict post-referral 12-month averages of these stratified estimates.

## eReferences

1. Meinhofer A, Chandra N, Byanova D, Keyes KM. Foster Care and Health in Medicaid-Enrolled Children Experiencing Parental Opioid Use Disorder. *JAMA Network Open*. 2024;7(5):e2410432-e2410432.

2. Wing C, Simon K, Bello-Gomez RA. Designing Difference in Difference Studies: Best Practices for Public Health Policy Research. *Annual Review of Public Health*. 2024;39(1):453-469. doi:10.1146/annurev-publhealth-040617-013507

3. Wing C, Freedman SM, Hollingsworth A. *Stacked Difference-in-Differences*. National Bureau of Economic Research; 2024. Accessed November 8, 2024. https://www.nber.org/papers/w32054

© 2025 Connell CM et al. *JAMA Health Forum*.
