# Supplementary material for: Role of Child Welfare in Detection and Treatment of Early Childhood Developmental Concerns
Source: JAMA Health Forum. 2025 Oct 24;6(10):e254554. doi: 10.1001/jamahealthforum.2025.4554 (PMC12552921; doi:10.1001/jamahealthforum.2025.4554)
Supplement: Supplement 2. — Data Sharing Statement [file jamahealthforum-e254554-s002.pdf]

Data Sharing Statement

Connell. Role of Child Welfare in Detection and Treatment of Early Childhood Developmental Concerns. *JAMA Health Forum*. Published October 24, 2025.
doi:10.1001/jamahealthforum.2025.4554

Data
Data available: No

Additional Information
Explanation for why data not available: Accessed via business associate agreement that does not permit data sharing
